# Treatment of Advanced Non-Small Cell Lung Cancer with RET Fusions: Reality and Hopes

**DOI:** 10.3390/ijms24032433

**Published:** 2023-01-26

**Authors:** Danilo Rocco, Luigi Sapio, Luigi Della Gravara, Silvio Naviglio, Cesare Gridelli

**Affiliations:** 1 Department of Pulmonary Oncology, AORN dei Colli Monaldi, 80131 Naples, Italy; 2 Department of Precision Medicine, University of Campania “Luigi Vanvitelli”, 80138 Naples, Italy; 3 Department of Experimental Medicine, University of Campania “Luigi Vanvitelli”, 80138 Naples, Italy; 4 Division of Medical Oncology, ‘S.G. Moscati’ Hospital, 83100 Avellino, Italy

**Keywords:** NSCLC, RET fusion, TKI, drug resistance

## Abstract

RET-selective tyrosine kinase inhibitors (TKIs) selpercatinib and pralsetinib have revolutionized the landscape of RET-positive (RET+) advanced non-small cell lung cancer (NSCLC) treatment, thanks to their efficacy and safety profiles. This class of medications currently represents the standard of care for both naïve and patients that have not received selective RET-TKIs in the first-line setting. However, we presently lack a satisfactory understanding of resistance mechanism developing after selective RET-TKIs usage, as well as a specific treatment for patients progressing on selpercatinib or pralsetinib. Chemotherapy ± immunotherapy is considered as a recommended subsequent second-line regimen in these patients. Therefore, it is of paramount importance to better define and understand the resistance mechanisms triggered by RET-TKIs. With this in mind, the present review article has been conceived to provide a comprehensive overview about RET+ advanced NSCLC, both from a therapeutic and molecular point of view. Besides comparing the clinical outcome achieved in RET+ advanced NSCLC patients after multikinase inhibitors (MKIs) and/or RET-selective TKIs’ administration, we focused on the molecular mechanisms accountable for their long-term resistance. Finally, a critical perspective on many of today’s most debated issues and concerns is provided, with the purpose of shaping the possible pharmacological approaches for tomorrow’s therapies.

## 1. Introduction

According to the most recent GLOBOCAN (Global Cancer Observatory) statistics, 19.3 million cancer cases were recorded in 2020, with 9.9 million cancer-related deaths. Lung cancer accounted for 2,206,771 diagnoses and for 1,796,144 deaths, representing the second most commonly diagnosed neoplasm in both females (after breast cancer) and males (after prostate cancer). Moreover, lung cancer stands as the leading cause of cancer-related death in males, and the second one in females (after breast cancer) [1].

More recent data from the American Cancer Society have further confirmed this trend. In 2022, 1,918,030 cancer cases and 609,360 cancer deaths are projected to occur in US. The estimated number of lung cancer cases is 236,740, with 130,180 estimated deaths. Therefore, lung cancer is projected to remain the second most commonly diagnosed neoplasm in US females (after breast cancer) and males (after prostate cancer). Conversely, lung cancer is projected to become the leading cancer-related cause of death in both males and female for the current year [2,3].

The median age at diagnosis for lung cancer patients is about 70 years old; at diagnosis, roughly 50–60% of patients are already metastatic (stage IV), 20–25% of patients present a locally advanced disease (stage III), and 20–25% of patients present an early-stage disease (stage I/II) [4,5,6].

Lung cancer is classified in two main histological types: NSCLC and SCLC (small-cell lung cancer); NSCLC accounts for approximately 85% of all lung cancer cases, while SCLC for the remaining 15% [7,8,9,10]. NSCLC is further divided in three distinct subtypes: adenocarcinoma (accounting for approximately 40–50% of all NSCLC cases), squamous cell carcinoma (20–30% of all NSCLC cases), and large cell carcinoma/not otherwise specified (10–20% of all NSCLC cases) [11,12,13].

While gene mutations can be found in both non-squamous and squamous NSCLC, as of today, selectively targetable mutations that play a key role in NSCLC growth and progression are mainly found in the non-squamous subtype (oncogene-addicted NSCLC) [14,15,16,17]. With reference to non-squamous NSCLC arising in Western patients, *KRAS* (Kirsten rat sarcoma viral oncogene homolog) gene mutations can be found in about 10–15% of patients, *EGFR* (epidermal growth factor receptor) in approximately 10–15%, *ALK* (anaplastic lymphoma kinase) in 3–5%, *BRAF* (v-raf murine sarcoma viral oncogene homolog B1) in 2–3%, *ROS1* (c-ros oncogene 1) in 1–2%, *RET* (rearranged during transfection) in 1–2%, *MET* (MET proto-oncogene) in 1–2%, and *NTRK* (neurotrophic receptor tyrosine kinase 1) in 0.1–1% of patients [18,19,20,21,22].

The approval of both targeted and immune therapy has revolutionized NSCLC management over past decades, providing remarkable therapeutic progress [23]. Biological therapies are usually administered alone or in combination with canonical chemotherapy [24]. Nearly all the above-mentioned mutations are currently druggable targets, while specific related inhibitors have been developed and tested especially in advanced NSCLC patients [25]. However, while there is no doubt that these advances have improved survival outcomes of advanced NSCLC, biological drugs provide a therapeutic benefit just for a small and specific subset of patients [26,27]. Moreover, acquired drug resistance often occurred in these patients, demanding additional medications [28,29].

Herein, we provide a comprehensive overview about the current treatment options for advanced NSCLC patients with RET fusions. Starting from pathogenesis characterization and epidemiological analysis of RET fusions in advanced NSCLC, we will subsequently compare the clinical outcome achieved after MKIs and/or RET-selective TKIs’ administration, using both retrospective and prospective trials’ results. Moreover, we will also focus our attention on the molecular mechanisms accountable for their long-lasting resistance. Finally, a critical investigation of the current related issues and concerns will be provided with the purpose of recognizing possible pharmacological approaches for anticancer therapies of tomorrow.

## 2. Canonical and Aberrant RET Signaling: Pathogenesis and Epidemiology in Advanced Non-Small Cell Lung Cancer

The *RET* gene is localized on chromosome 10 and encodes for RET-RTK (RET receptor tyrosine kinase). Unlike other RTK, RET is marked by four cadherin-like extracellular domains and 16 cysteine residues within its amino acid sequence [30]. Physiologically, RET-RTK is activated through multiple events, which include binding of Ca_2_^+^ ions to the cadherin-like domains, as well as recognition between GFLs (glial-derived neurotrophic factor ligands) and GFRs (glial-derived neurotrophic factor family receptors) [31]. As a result of its activation, RET-RTK undergoes homodimerization and autophosphorylation, leading to activation of downstream signaling cascades [32]. Depending on the phosphorylation site, RET-RTK enables different pathways, such as PI3K/AKT, RAS/RAF/MEK/ERK, JAK2/STAT3, and PLC-γ, which in turn affect cell proliferation, survival, and differentiation [33].

RET expression is usually temporally and spatially regulated in both embryonic and adult tissues. A striking example is the kidney expression, where RET signaling is considered critical for normal development at the embryonic stage, while it was largely absent in the adult organ [34]. Apart from playing an important role in embryonic kidney development, RET expression is also required for the proper development, maturation, and maintenance of the autonomic and enteric nervous systems (ENS), as well as spermatogenesis and determination of spermatogonial stem cell fate [35,36]. 

Starting from the first detection of an oncogenic RET alteration in papillary thyroid carcinomas, multiple genetic rearrangements have been identified in solid tumors over the years. Typically, pathological intrachromosomal rearrangements arise from juxtaposition of two otherwise independent genes, resulting from inversions or translocations, transcriptional reading of adjacent genes, or splicing of pre-mRNA sequences [33]. *KIF5B* (kinesin family member 5B) (~70% of cases) and *CCDC6* (coiled-coil domain containing 6) (~20% of cases) represent the main RET fusion partners in malignant tumors, even though additional ones such as *NCOA4* (nuclear receptor coactivator 4), *TRIM33* (tripartite motif containing 33), *MYO5C* (myosin VC), and *EPHA5* (enables ephrin receptor A5) have been detected so far [37]. RET fusions usually cause loss of the transmembrane domain, giving rise to a chimeric cytosolic protein, which in turn leads to development of an aberrant, ligand-independent and constitutively activated RET kinase domain. Despite the role of RET as a proto-oncogene was firstly described in 1985, RET fusions in advanced NSCLC were only identified in 2012 [38,39,40,41].

According to the most recent data, RET-rearranged advanced NSCLCs account for approximately 1–2% of all diagnoses, representing ~ 10.000 to 15.000 new cases every year. Advanced NSCLC patients with RET fusions are typically younger, never/light-smoker females with adenocarcinomas, who present an increased risk of central nervous system metastases [33,34,35,36,37]. Compared with other alternative oncogenic aberrations recognized in NSCLC, RET fusion-positive lung carcinomas had more poorly differentiated tumors, suggesting that this kind of genetic aberration defines a unique molecular and clinicopathological subtype [38].

## 3. Early Attempts at Target Therapy for RET-Rearranged Advanced Non-Small Cell Lung Cancer: Multikinase Inhibitors

In agreement with the literature data, while RET-rearranged advanced NSCLC seems to be responsive to classic platinum-based cytotoxic therapy, it appears to be scarcely sensitive to immunotherapy in the form of immune checkpoint inhibitors (being a “cold” tumor with a low tumor mutational burden). In this vein, RET+ NSCLC data are consistent with those coming from other oncogene-addicted NSCLCs, such as ALK+, EGFR+, and ROS1+ [38,39,40,41,42].

Therefore, starting from 2012, the search for a targeted therapy that could grant superior efficacy results and better tolerability than chemotherapy was begun. In early trials, the choice fell on MKIs, namely drugs that inhibit RET-RTK alongside with other RTKs and/or kinases such as VEGFR (vascular endothelial growth factor receptor), BRAF, ALK, and EGFR [43,44,45]. Both retrospective and prospective trials were conducted, investigating several different agents: cabozantinib, an anti-RET, MET, AXL (tyrosine-protein kinase receptor UFO), VEGFR, FLT3 (FMS-like receptor tyrosine kinase-3), and KIT (KIT Proto-Oncogene); sorafenib, an anti-RET, BRAF, VEGFR, PDGFR (platelet derived growth factor receptor alpha), KIT, FLT3, and FGFR (fibroblast growth factor receptor); vandetanib, an anti-VEGFR, EGFR, and RET; lenvatinib, an anti-VEGFR, FGFR, PDGFR, RET, and KIT; sunitinib, an anti-PDGFR, VEGFR, KIT, RET, CSF-1R (colony stimulating factor 1 receptor), and FLT3; alectinib, an anti-ALK and RET; ponatinib, an anti-VEGFR, PDGFR, FGFR, EPH-RTK, KIT, RET, TIE2 (TEK receptor tyrosine kinase), and FLT3; nintedanib, an anti-PDGFR, FGFR VEGFR, FLT3, and RET; regorafenib, an anti-RET, BRAF, VEGFR, KIT, PDGFR, FGFR, TIE2, and EPH-RTK [46] (Table 1). With reference to retrospective trials, cabozantinib, vandetanib, lenvatinib, sorafenib, sunitinib, alectinib, ponatinib, regorafenib, and nintedanib were investigated in a global study by Gautschi et al. (GLORY database). Basically, 53 pretreated (median number of lines of therapy: three, ranging from one to eight) RET+ advanced NSCLC patients received an MKI-treatment, and response data were available in 50 patients: 19 patients receiving cabozantinib with a DCR (disease control rate) of 63%, an mPFS (median progression free survival) of 3.6 months, and an mOS (median overall survival) of 4.9 months; 11 patients receiving vandetanib with a DCR of 45%, an mPFS of 2.9 months, and an mOS of 10.2 months; nine patients receiving sunitinib with a DCR of 55%, an mPFS of 2.2 months, and an mOS of 6.8 months; 2 patients receiving sorafenib achieving two SDs (stable disease); two patients receiving alectinib achieving two PDs (progression of disease); two patients receiving lenvatinib achieving one PR (partial response) and one PD; two patients receiving nintedanib achieving one CR (complete response) and one SD; two patients receiving ponatinib achieving two SDs; and one patient receiving regorafenib achieving a PD [47].

Vandetanib was also retrospectively evaluated in a paper by Platt et coll., in which three pretreated RET+ NSCLC patients received this compound. However, no objective responses were reported [48].

In the same vein, alectinib activity in pretreated RET+ advanced NSCLC patients was assessed in two case series by Lin et al. (four patients) and by Ribeiro et al. (four patients), reporting one PR and one SD, one SD and one PMR (partial molecular response), respectively [49,50].

On the other hand, with reference to prospective trials, cabozantinib was investigated in an open-label phase II trial by Drilon et al., where 26 naïve and pretreated RET+ advanced NSCLC patients were enrolled, and 25 patients were evaluable; 23% of patients received cabozantinib as a first-line treatment, 50% of patients received cabozantinib as a second-line treatment, and 27% of patients received cabozantinib as a third or further-line treatment (all pretreated patients received prior chemotherapy regimens, but no prior RET-TKI therapies). The reported ORR (overall response rate) was 28%, the DCR was 100%, the mPFS was 5.5 months, and the mOS was 9.9 months; a better trend (not statistically significant) in terms of survival was reported for naïve patients. TRAEs (treatment related adverse events) of any grade were reported in 96.2% of treated patients (hypothyroidism, elevated liver enzymes, diarrhea, and palmar plantar erythrodysesthesia being the most common ones); the most common grade 3 TRAEs were lipase elevation, liver enzyme elevation, and thrombocytopenia. It is noteworthy to mention that 73% of treated patients required a dose reduction due to cabozantinib-related TRAEs, mainly due to palmar plantar erythrodysesthesia, fatigue, and diarrhea; 8% of treated patients discontinued cabozantinib following TRAEs [51]. Cabozantinib was also assessed in a phase I trial by Nokihara et al., in which two RET+ advanced NSCLC patients were enrolled, reporting an ORR of 50% [52].

Similarly, sorafenib was evaluated in a prospective phase II study by Horiike et al., enrolling three pretreated RET+ advanced NSCLC patients. Two out of the three patients experienced rapid PD (time to progression 18 and 43 days, respectively), and one patient experienced an SD; in this last patient, two dose reductions were needed following grade 3 palmar plantar erythrodysesthesia and a grade 3 infection. The reported ORR for this study was 0%, while the DCR was 33.3% [53].

In the same vein, vandetanib monotherapy was evaluated in two phase II prospective trials by Lee et al. and Yoh et al., respectively. The former trial enrolled 18 heavily pretreated (72% of treated patients had already received ≥ two previous lines of chemotherapy) RET+ advanced NSCLC patients; 17 patients presented available data. Three patients presented a PR, and eight patients presented an SD (ORR: 18%, DCR: 65%); the mPFS was 4.5 months, while the mOS was 11.6 months. With reference to the safety and tolerability profile, five cases of grade 3 TRAEs were reported (hypertension, liver enzyme elevation, and QT prolongation), and dose reductions were required in four patients [54]. Almost 19 pretreated RET+ advanced NSCLC patients were enrolled in the latter study, achieving an ORR of 47%, a DCR of 90%, and an mPFS of 4.7 months. Grade 3 or 4 hypertension was reported in 58% of treated patients, acneiform rash was reported in 16% of treated patients, while QT prolongation and diarrhea were reported in 11% of treated patients; 21% of treated patients experienced a treatment-related drug discontinuation, while 53% of treated patients had to reduce vandetanib dosage [55]. On the other hand, Hida et al. conducted a phase II prospective study investigating lenvatinib in mostly pretreated (92% of enrolled patients) RET+ advanced NSCLC patients. Overall, 25 patients received lenvatinib monotherapy, reporting an ORR of 16%, a DCR of 76%, an mPFS of 7.3 months, and a 2-year OS of 54.5%; the toxicity profile, however proved to be unfavorable; ≥grade 3 TRAES: 92%, TRAEs leading to dose reduction: 64%, TRAEs leading to drug interruption: 76%, TRAEs leading to drug discontinuation: 24%; three patients died following lenvatinib treatment [56].

In summary, MKI therapy for RET+ advanced NSCLC patients proved to be associated with modest efficacy results and with serious TRAEs. The most likely reason behind these results lies in the not-selective inhibition of the RET-RTK, while another factor could be represented by patients’ selection, as almost every patient in the above-mentioned trials was a pretreated one [57,58]. As a result, none of these drugs received FDA (US Food and Drug Administration) approval in this setting.

**Table 1:** Data coming from retrospective and prospective trials involving MKIs for the treatment of advanced RET+ NSCLC patients.

Authors	Type of Trial/Phase	Drug	Patients	Efficacy Results	Safety Profile
Gautschi et al. [47]	Retrospective	Cabozantinib	19	DCR: 63% mPFS: 3.6 months mOS: 4.9 months	Not Available (N/A)
Drilon et al. [51]	Prospective Phase II	Cabozantinib	25	ORR: 28% DCR: 100% mPFS: 5.5 months mOS: 9.9 months	TRAEs leading to dose reductions: 73% TRAEs leading to drug discontinuation: 8%
Nokihara et al. [52]	Prospective Phase I	Cabozantinib	2	ORR: 50%	N/A
Gautschi et al. [47]	Retrospective	Vandetanib	11	DCR: 45% mPFS: 2.9 months mOS: 10.2 months	N/A
Platt et al. [48]	Retrospective	Vandetanib	3	DCR: 0%	N/A
Lee et al. [54]	Prospective Phase II	Vandetanib	17	ORR: 18% DCR: 65% mPFS: 4.5 months mOS: 11.6 months	Grade 3 TRAEs: 29.4% TRAEs leading to dose reductions: 23.5%
Yoh et al. [55]	Prospective Phase II	Vandetanib	19	ORR: 47% DCR: 90% mPFS: 4.7 months	TRAEs leading to dose reductions: 53% TRAEs leading to drug discontinuation: 21%
Gautschi et al. [47]	Retrospective	Sunitinib	9	DCR: 55% mPFS: 2.2 months mOS: 6.8 months	N/A
Gautschi et al. [47]	Retrospective	Sorafenib	2	DCR: 100%	N/A
Horiike et al. [53]	Prospective Phase II	Sorafenib	3	ORR: 0% DCR: 33.3%	TRAEs leading to dose reductions: 73%
Lin et al. [49]	Retrospective	Alectinib	4	DCR: 100%	N/A
Ribeiro et al. [50]	Retrospective	Alectinib	4	DCR: 50%	N/A
Gautschi et al. [47]	Retrospective	Alectinib	2	DCR: 0%	N/A
Gautschi et al. [47]	Retrospective	Lenvatinib	2	DCR: 50%	N/A
Hida et al. [56]	Prospective Phase II	Lenvatinib	25	ORR: 16% DCR: 76% mPFS: 7.3 months	≥ grade 3 TRAES: 92% TRAEs leading to dose reductions: 64% TRAEs leading to drug interruption: 76% TRAEs leading to drug discontinuation: 24%
Gautschi et al. [47]	Retrospective	Nintedanib	2	DCR: 100%	N/A
Gautschi et al. [47]	Retrospective	Ponatinib	2	DCR: 100%	N/A
Gautschi et al. [47]	Retrospective	Regorafenib	1	DCR: 0%	N/A

## 4. Current Standard of Care for RET-Rearranged Advanced Non-Small Cell Lung Cancer: RET-Selective Tyrosine Kinase Inhibitors 

**Figure 1:**
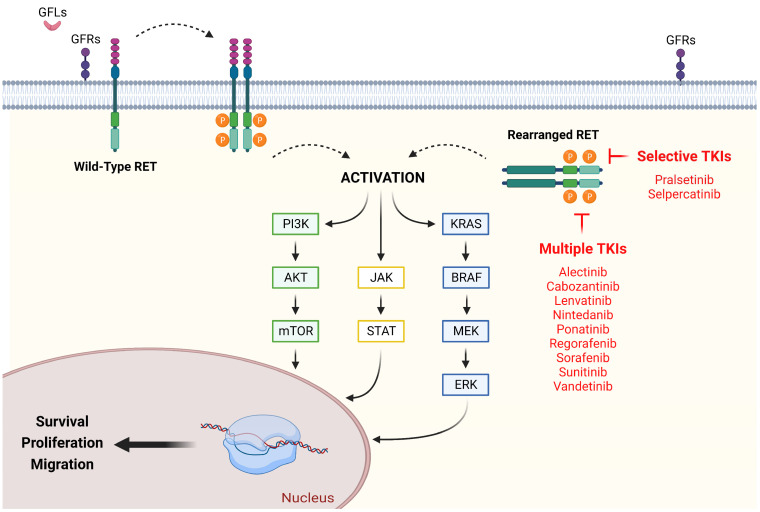
RET signaling in NSCLC: existing therapeutic strategies for its neutralization. Typically, RET activation requires a pre-binding between GFLs and GFRs, as well as between Ca_2_^+^ and cadherin-like domains, which in turn allow homodimerization and autophosphorylation of the cytosolic tyrosine kinase domains. Genetic rearrangements of RET cause loss of transmembrane domain, resulting in a ligand-independent and constitutively activated chimeric cytosolic protein. Once active, RET switch-on different pathways, such as P13K/AKT, RAS/MAPK, and JAK/STAT, promoting survival, proliferation, and migration. Multiple and selective TKIs constrain the above vicious circle by competing with ATP for binding. AKT (protein kinase B); BRAF (v-raf murine sarcoma viral oncogene homolog B1); ERK (extracellular signal-regulated kinase); GFLs (glial-derived neurotrophic factor ligands); GFRs (glial-derived neurotrophic factor family receptors); JAK (Janus Kinase); KRAS (Kirsten rat sarcoma); MEK (mitogen-activated protein kinase kinase), mTOR (mammalian target of rapamycin); PI3K (PhosphatidylInositol 3-Kinase); RET (rearranged during transfection); STAT (signal transducer and activator of transcription). Created with BioRender.com (accessed on 21 December 2022).

Learning from shortcomings and limitations of MKIs, new RET-selective TKIs were developed and assessed in both pretreated and naïve RET+ advanced NSCLC patients (Figure 1). These trials displayed remarkably successful results, receiving for two RET-specific TKIs (selpercatinib and pralsetinib) FDA approval and ASCO (American Society of Clinical Oncology) guidelines recommendation, either naïve patients or second-line setting for patients who have not received a selective RET-TKI in the first-line setting [59,60].

Selpercatinib was investigated in the open-label phase I/II LIBRETTO-001 trial, in which 105 pretreated (with at least one platinum doublet chemotherapy) and 39 naïve RET+ advanced NSCLC patients received selpercatinib in monotherapy. With reference to pretreated patients, the median number of previous treatments was three (ranging from one to 15 lines); 55% of treated patients had already received immune checkpoint inhibitors, and 48% of treated patients had already received MKI; at data cut-off, selpercatinib managed to provide extremely favorable results: the ORR was 64%, the DCR was 93%, and the mPFS was 16.5 months. With respect to naïve patients, even more remarkable results were reported, reaching an ORR of 85% and a DCR of 95%, while the mPFS was still not reached. The safety and tolerability profile proved to be manageable: grade ≥ 3 TRAEs were reported in 28% of treated patients, mainly hypertension (14% of treated patients) and liver enzymes elevation (13% and 10% of treated patients for ALT and AST, respectively). TRAEs leading to selpercatinib dose reduction were reported in 30% of treated patients, while TRAEs leading to selpercatinib interruption were reported in 2% of treated patients [61]. After a longer follow-up, an expanded data set of 316 patients (247 pretreated and 69 naïve ones) was provided by the authors, further confirming the excellent performance of selpercatinib. In pretreated patients, the ORR was 61%, the DCR was 95%, the mPFS was 24.9 months, and the 3-year OS rate was 58.5%. In naïve patients, the ORR was 84%, the DCR was 93%, the mPFS was 22.0 months, and the 3-year OS rate was 57.1%. The safety and tolerability profile proved to be consistent with the previous signals, with grade ≥ 3 TRAEs reported in 38.6% of treated patients [62].

On the other hand, pralsetinib was assess in the open-label phase I/II ARROW study, in which 114 RET+ advanced NSCLC patients (87 pretreated and 27 naïve ones) received pralsetinib in monotherapy. The 87 pretreated patients received a median of two previous lines of treatment; 45% of treated patients had already received immune checkpoint inhibitors, and 26% of treated patients had already received MKI; the reported ORR was 61%, the DCR was 91%, and the mPFS was 17.1 months. Superior results were associated with naïve patients, with an ORR of 70%, a DCR of 85%, and an mPFS of 9.1 months. Grade ≥ 3 TRAEs occurred in 48% of treated patients, mainly neutropenia (18% of treated patients) and hypertension (11% of treated patients); 38% of treated patients experienced TRAEs leading to dose reductions, and 6% of treated patients experienced TRAEs leading to drug discontinuation [63]. These results were confirmed after an extended follow-up including 211 patients (136 pretreated and 75 naïve ones). Pretreated patients obtained an ORR of 59%, a DCR of 90%, an mPFS of 16.5 months, and a 12-month OS rate of 72%; naïve patients obtained an ORR of 72%, a DCR of 91%, an mPFS of 13.0 months, and a 12-month OS rate of 82%. No new safety signals were reported, with 20% of treated patients reporting grade ≥ 3 TRAEs and 7% of treated patients discontinuing pralsetinib due to TRAEs [64].

## 5. Resistance Mechanisms to RET-Selective Tyrosine Kinase Inhibitors and Potential Strategies to Overcome Tumor Adaptation 

Compared to MKIs, RET-selective TKIs manage to grant far superior efficacy and safety results. However, resistance to these agents eventually emerges in all treated patients. In this vein, as of today, no specific treatment is available for RET-selective TKI-resistant patients, and ASCO guidelines recommend standard chemotherapy (±immunotherapy) in this setting [60]. Thus, understanding the mechanisms behind selpercatinib/pralsetinib resistance is crucial to develop a more specific treatment for this subset of patients, reserving a more toxic and less tolerable therapy, such as chemotherapy, for a further line of treatment. According to the literature data, we can classify resistance mechanisms to TKI treatment according to the involved area of the kinase (gatekeeper mutations versus solvent front mutations) and according to whether or not the original mutated kinase pathway is involved (on-target resistance versus off-target resistance). With reference to resistance mutations arising in RET-selective TKI-resistant patients, even though data are currently scarce given the recent development of these agents, on-target resistance mutations seem to be less frequent than in other oncogene-addicted NSCLCs (i.e., EGFR+, ALK+, ROS1+, etc.) and mainly represented by G810 R/S/C/V RET solvent front mutation. On the other hand, off-target resistance mechanisms seem to be more frequent, being principally represented by MET and KRAS amplification [65].

In a recent paper, Solomon et al. described for the first time G810 R/S/C RET solvent front resistance mutations analyzing ctDNA from two RET+ advanced NSCLC patients progressing on selpercatinib; these findings were further confirmed via postmortem biopsies [66,67,68].

In a recent 2020 paper, Lin and coworkers assessed 20 tissue and/or cfDNA biopsies from 18 RET+ advanced NSCLC patients after RET-selective TKI treatment (10 patients received selpercatinib, seven patients received pralsetinib, and one patient received pralsetinib and selpercatinib). The RET G810 solvent front resistance mutation was reported in two patients, three patients presented MET amplification as an off-target resistance mechanism, and one patient presented KRAS amplification as an off-target resistance mechanism [69]. Similarly, in a post-hoc analysis from the ARROW study, RET solvent front resistance mutations (G810 and L730) were reported in approximately 10% of analyzed ctDNA specimens [70].

Lastly, in an extremely recent 2022 study by Rosen et coll., these results were further confirmed. Almost 13 RET+ advanced NSCLC patients progressing on selpercatinib underwent plasma ± tissue biopsy, and eight patients presented evaluable results: two patients presented RET G810 solvent front resistance mutations, while one patient presented MET amplification [71].

The main current strategy under investigation in order to treat RET G810+ patients is represented by the development of selective RET-TKI, capable of being effective against this mutation. Presently, TPX-0046 (a RET/SRC inhibitor) is one of the most promising candidates towards RET G810 mutation. After demonstrating preclinical activity, it is now being evaluated in phase I testing in RET+ solid tumor patients (NCT04161391) [72]. 

On the other hand, with respect to KRAS or MET amplified RET-selective TKI-resistant patients, the most interesting strategy is represented by TKI combination treatments. Early anecdotal evidence in this sense is coming from a small subgroup analysis from the LIBRETTO-001 trial. Essentially, four MET amplified (in one patient, MET amplification was already present at baseline) selpercatinib-resistant patients received selpercatinib + crizotinib (a multitargeted tyrosine kinase inhibitor originally developed to inhibit c-MET) following post-selpercatinib progression, with responses lasting 3.5 months, 10 months, 1.5 months (the patient died for unrelated cardiac causes), and 4 weeks (the patient developed unrelated colitis and suspended the combination) [73]. 

Both the above-mentioned strategies will need further and larger tested to investigate and validate these findings. 

## 6. Additional Molecular Insight on Tyrosine Kinase Inhibitors Resistance in RET-Positive NSCLC 

As highlighted by clinical results, understanding the molecular mechanisms by which RET+ NSCLCs become unresponsive to TKIs is crucial in order to provide a more effective therapeutic option in the foreseeable future.

Regrettably, due to the limited number of RET fusion-driven NSCLC models, as well as RET-TKI resistant ones, the molecular characterization of this adaptive tumor behavior is far from over. Besides the aforementioned patient-derived genomic mutations and amplification, only a handful of preliminary studies have been conducted to figure out the RET-TKI resistance in NSCLC. In this respect, an exhaustive overview of the existing knowledge is provided below.

Using ponatinib as an MKI to counteract RET fusion-positive (CCDC6-RET) LC-2/ad lung adenocarcinoma cell growth, Nelson-Taylor and colleagues demonstrated that inhibition of RET phosphorylation is accompanied by ERK1/2 and AKT inactivation [74]. Interestingly, after having developed two distinct ponatinib-resistant LC-2/ad cell lines, PR1 and PR2, they observed that while ponatinib was still capable of down-regulating RET phosphorylation in the unresponsive models, its impact on both ERK1/2 and AKT signaling was negligible. Assuming an acquired bypass signaling that drove PI3K/AKT and RAS/RAF/MEK/ERK activation, independent of RET signaling in PR1 and PR2 cells, they subsequently performed next-generation sequencing aimed at identifying differentially expressed genes and/or mutations between parental and resistant LC-2/ad cells. Nelson-Taylor and co-workers recognized a single base pair substitution in *NRAS* gene encoding for p.Q61K mutant in PR1, but not in the parental LC-2/ad or PR2 cells. Interestingly, siRNA knockdown of *NRAS* decreased cell proliferation only in PR1, while no effects were detected in both PR2 and parental ones. Unlike the previous ponatinib-resistant cells, PR2 showed activation of wild-type EGFR and AXL signaling instead. Besides affecting proliferation in a more effective way, the use of specific EGFR or AXL inhibitors decreased phospho-ERK1/2 and phospho-AKT only in PR2 cells.

To corroborate the relevance of EGFR signaling in triggering resistance to RET inhibitors, Chang and colleagues reported how treatment with EGF (epidermal growth factor) dose-dependently reduced the sensitivity to RET inhibitors in LC-2/ad cells [75]. According to Nelson-Taylor findings, the employed RET inhibitors (sunitinib, E7080, vandetanib, and sorafenib) reduced RET phosphorylation, suppressing activation of AKT and ERK1/2. Remarkably, no RET inhibitors were able of diminishing phosphorylation of AKT and ERK1/2 in the presence of EGF. Apart from re-sensitizing LC-2/ad cells to RET inhibitors, even in the presence of EGF, the concomitant presence of EGFR inhibitors prevented AKT and ERK1/2 phosphorylation. They also showed how co-culture with HUVEC endothelial cells caused a heterogeneous response to RET inhibitors by activating bypass survival signals of EGFR, opening up a new frontier about the role of the tumor microenvironment in RET-TKIs’ resistance.

The potential pivotal role of ERK1/2 in mediating adaptive resistance to TKI in RET fusion-positive tumor cells was supported by Ramen’s findings [76]. Despite achievement in CCDC6-RET-rearranged thyroid cancer cells, they found that exposure to either cabozantinib (a non-selective RET-TKIs) or BLU6864 (a selective RET-TKIs) is associated with rapid inhibition of ERK1/2 signaling. However, as observed in NSCLC, prolonged exposure to these compounds decreased RET phosphorylation and rebounded ERK1/2 activation. In an effort to identify signaling pathways responsible for mediating adaptive resistance to TKIs, the authors performed proteome profiling of phosphotyrosine using mass spectrometry. The achieved results revealed an overactivation of JAK2/STA3 in response to TKIs’ administration, as a downstream of FGFR signaling. Fascinatingly, combined treatment with RET and FGFR inhibitors effectively abrogated adaptive resistance and led to a decrease in ERK1/2 signaling. This outcome was also corroborated by lentiviral infection and CRISPR/Cas9 gene editing, targeting FGFR1 for genetic inactivation.

Moving to a different RET fusion, the involvement of AKT and ERK1/2 signaling in the adaptive resistance to TKIs is quite intricate. In this regard, Schubert and colleagues established three patient-derived NSCLC cell lines, two harboring KIF5B-RET fusion (CUTO22 and CUTO32) and one containing an EML4-RET rearrangement (CUTO42) [77]. They observed that CUTO22 and CUTO42 were responsive to both selective and non-selective RET inhibitors, whereas CUTO32 was markedly resistant. Even though TKIs were capable of reducing RET phosphorylation in the sensitive cells, CUTO22 displayed no reduction in phospho-AKT. Strangely, the employment of rapamycin (mTOR) inhibitor omipalisib conferred a resistant phenotype in CUTO22. The RET TKIs-resistant CUTO32 cells did not show changes in either ERK1/2 or AKT phosphorylation, despite successful RET inhibition. Besides raising doubts about the central role of these pathways in developing RET-TKI unresponsiveness, Schubert and co-workers recognized additional resistant signatures in CUTO32, including PLK1 (polo-like kinase 1), Aurora kinase, MET, and MYC.

The potential engagement of the MYC pathway in RET-fusion NSLCLs is also discussed by Hayashi’s group in their recent study [78]. Performing transcriptomic analysis of lung tumors and cell lines with RET alterations, they identified significant activation of MYC-associated transcriptional signatures. Although MYC activation was suppressed by treatment with cabozantinib, no experiments were performed in order to explore its role in developing resistance, however.

Considering the attention paid towards combination therapy in NSCLC instead, Fujimura searched for a compatible agent that could be used in a mixture with alectinib, a small molecule agent with RET kinase inhibitory activity, to enhance its antitumor effects [79]. Using two distinct RET-fusion positive NSCLC cells, LC-2/ad (CCDC6-RET) and Ba/F3-KIF5B-RET (KIF5B-RET), they detected the highest synergistic effect combining alectinib with palbociclib, a CDK4/6 (cyclin-dependent kinase 4 and 6) inhibitor. Apart from down-regulating well-known targets of both alectinib and palbociclib, the authors did not report any additional information about the combination molecular mechanisms, as well as palbociclib-mediated effects in RET-TKI resistance. 

In order to overcome RET preclinical models’ limitations, in silico analysis has been performed to predict which RET on-target alterations confer TKI resistance. Using this approach, Repetto and collaborators have recently investigated the potential resistance mechanisms triggered towards TPX-0046 compounds [80]. As a new generation macrocyclic RET/SRC-inhibitor, TPX-0046 has proved to be effective against a range of RET fusions and mutations, including solvent front mutations (i.e., RET G810 substitutions) [81]. Based on the achieved results, the authors predicted the inability of TPX-0046 to bind RET when bulky hydrophobic gatekeeper mutations occurred within V804L/M, and to a lesser extent, L881F or G810S + S891L.

## 7. Conclusions

RET-selective TKIs (selpercatinib and pralsetinib) have revolutionized the landscape of RET+ advanced NSCLC treatment thanks to their efficacy and safety profiles, and currently represent the standard of care for both naïve patients and patients that have not received a selective RET-TKI in the first-line setting. However, we presently lack a specific treatment for patients progressing after selpercatinib or pralsetinib therapy, who are currently managed with chemotherapy ± immunotherapy as a subsequent line regimen. In this vein, it is of paramount importance to define and understand the resistance mechanisms of these patients (i.e., RET G810 mutation, KRAS/MET amplifications, and potentially new ones), in order to develop more tailored agents. 

Whilst the ongoing clinical trials propose new chances for treating RET+ advanced NSCLC patients, larger and differential studies are required in order to identify the right therapeutic regimens. In this respect, a sequential resistance mechanism-specific algorithm, analogous to those in place and further developing for EGFR+ or ALK+ NSCLC, could represent an ambitious but optimal goal [82,83]. With reference to EGFR+ advanced NSCLC patients, in the ongoing ORCHARD trial, ~ 150 patients experiencing progression of disease after upfront osimertinib (mutant-selective EGFR inhibitor) will undergo a post-progression biopsy in order to be assigned to one of three arms (A, B, or C).

While arm C is the observational one, and B enrolls patients with a non-specifically targetable secondary mutation mechanisms or without a secondary mutation mechanism, arm A enrolls patients with a specifically-targetable secondary mutation mechanism, assigning them to specific treatments. Osimertinib plus savolitinib (a MET-TKI) is the treatment of choice if the secondary mutation mechanism is a MET amplification; osimertinib plus gefitinib (an EGFR-TKI) is the treatment of choice if the secondary mutation mechanism is an EGFR mutation occurring in C797S; osimertinib plus necitumumab (an anti EGFR mAb) is the treatment of choice if the secondary mutation mechanism is an EGFR amplification; osimertinib plus alectinib is the treatment of choice if the secondary mutation mechanism is an ALK mutation; and osimertinib plus selpercatinib is the treatment of choice if the secondary mutation mechanism is a RET mutation [84].

In this vein—with reference to RET+ advanced NSCLCs—patients progressing on selpercatinib or pralsetinib could receive a TPX-0046-like drug if a RET G810 solvent front resistance mutation is detected or a selpercatinib + crizotinib-like combination if a KRAS or MET amplification is found following re-biopsy.

More generally, a deeper molecular mechanisms characterization is required for patients experiencing RET-TKI resistance. The available data results about this sadly adaptive event are quite fragmented and complicated to predict, since intra- and inter-tumor heterogeneity may exist. Nevertheless, combining clinical and molecular findings still represents the only viable way to provide new hopes and perspectives for RET+ advanced NSCLC patients.

## Data Availability

Not applicable.

## References

[1] Sung H., Ferlay J., Siegel R.L., Laversanne M., Soerjomataram I., Jemal A., Bray F. (2021). Global Cancer Statistics 2020: GLOBOCAN Estimates of Incidence and Mortality Worldwide for 36 Cancers in 185 Countries. CA Cancer J. Clin..

[2] Siegel R.L., Miller K.D., Fuchs H.E., Jemal A. (2022). Cancer statistics, 2022. CA Cancer J. Clin..

[3] Oliver A.L. (2022). Lung Cancer: Epidemiology and Screening. Surg. Clin. N. Am..

[4] Thai A.A., Solomon B.J., Sequist L.V., Gainor J.F., Heist R.S. (2021). Lung cancer. Lancet.

[5] Thandra K.C., Barsouk A., Saginala K., Aluru J.S., Barsouk A. (2021). Epidemiology of lung cancer. Contemp. Oncol..

[6] Zappa C., Mousa S.A. (2016). Non-small cell lung cancer: Current treatment and future advances. Transl. Lung Cancer Res..

[7] Sher T., Dy G.K., Adjei A.A. (2008). Small cell lung cancer. Mayo Clin. Proc..

[8] Kenfield S.A., Wei E.K., Stampfer M.J., Rosner B.A., Colditz G.A. (2008). Comparison of aspects of smoking among the four histological types of lung cancer. Tob. Control..

[9] Noguchi M., Morikawa A., Kawasaki M., Matsuno Y., Yamada T., Hirohashi S., Kondo H., Shimosato Y. (1995). Small adenocarcinoma of the lung. Histologic characteristics and prognosis. Cancer.

[10] Socinski M.A., Obasaju C., Gandara D., Hirsch F.R., Bonomi P., Bunn P., Kim E.S., Langer C.J., Natale R.B., Novello S. (2016). Clinicopathologic Features of Advanced Squamous NSCLC. J. Thorac. Oncol..

[11] Molina J.R., Yang P., Cassivi S.D., Schild S.E., Adjei A.A. (2008). Non-small cell lung cancer: Epidemiology, risk factors, treatment, and survivorship. Mayo Clin. Proc..

[12] Ganti A.K., Klein A.B., Cotarla I., Seal B., Chou E. (2021). Update of Incidence, Prevalence, Survival, and Initial Treatment in Patients With Non-Small Cell Lung Cancer in the US. JAMA Oncol..

[13] Inamura K. (2017). Lung cancer: Understanding its molecular pathology and the 2015 WHO classification. Front. Oncol..

[14] Chan B.A., Hughes B.G. (2015). Targeted therapy for non-small cell lung cancer: Current standards and the promise of the future. Transl. Lung Cancer Res..

[15] Wang M., Herbst R.S., Boshoff C. (2021). Toward personalized treatment approaches for non-small-cell lung cancer. Nat. Med..

[16] Ferrara M.G., Di Noia V., D’Argento E., Vita E., Damiano P., Cannella A., Ribelli M., Pilotto S., Milella M., Tortora G. (2020). Oncogene-Addicted Non-Small-Cell Lung Cancer: Treatment Opportunities and Future Perspectives. Cancers.

[17] Zhang Y.-L., Yuan J.-Q., Wang K.-F., Fu X.-H., Han X.-R., Threapleton D., Yang Z.-Y., Mao C., Tang J.-L. (2016). The prevalence of *EGFR* mutation in patients with non-small cell lung cancer: A systematic review and meta-analysis. Oncotarget.

[18] Skoulidis F., Heymach J.V. (2019). Co-occurring genomic alterations in non-small-cell lung cancer biology and therapy. Nat. Rev. Cancer.

[19] Hirsch F.R., Scagliotti G.V., Mulshine J.L., Kwon R., Curran W.J., Wu Y.-L., Paz-Ares L. (2017). Lung cancer: Current therapies and new targeted treatments. Lancet.

[20] Chevallier M., Borgeaud M., Addeo A., Friedlaender A. (2021). Oncogenic driver mutations in non-small cell lung cancer: Past, present and future. World J. Clin. Oncol..

[21] Zhu Q.-G., Zhang S.-M., Ding X.-X., He B., Zhang H.-Q. (2017). Driver genes in non-small cell lung cancer: Characteristics, detection methods, and targeted therapies. Oncotarget.

[22] Yang S.-R., Schultheis A.M., Yu H., Mandelker D., Ladanyi M., Büttner R. (2020). Precision medicine in non-small cell lung cancer: Current applications and future directions. Semin. Cancer Biol..

[23] Yuan M., Huang L.-L., Chen J.-H., Wu J., Xu Q. (2019). The emerging treatment landscape of targeted therapy in non-small-cell lung cancer. Signal Transduct. Target. Ther..

[24] Herbst R.S., Morgensztern D., Boshoff C. (2018). The biology and management of non-small cell lung cancer. Nature.

[25] Chen R., Manochakian R., James L., Azzouqa A.-G., Shi H., Zhang Y., Zhao Y., Zhou K., Lou Y. (2020). Emerging therapeutic agents for advanced non-small cell lung cancer. J. Hematol. Oncol..

[26] Ye Z., Huang Y., Ke J., Zhu X., Leng S., Luo H. (2020). Breakthrough in targeted therapy for non-small cell lung cancer. Biomed. Pharmacother..

[27] Reck M., Rodríguez-Abreu D., Robinson A.G., Hui R., Csőszi T., Fülöp A., Gottfried M., Peled N., Tafreshi A., Cuffe S. (2016). Pembrolizumab versus Chemotherapy for PD-L1–Positive Non–Small-Cell Lung Cancer. N. Engl. J. Med..

[28] Meador C.B., Hata A.N. (2020). Acquired resistance to targeted therapies in NSCLC: Updates and evolving insights. Pharmacol. Ther..

[29] Frisone D., Friedlaender A., Addeo A., Tsantoulis P. (2022). The Landscape of Immunotherapy Resistance in NSCLC. Front. Oncol..

[30] Ibáñez C.F. (2013). Structure and Physiology of the RET Receptor Tyrosine Kinase. Cold Spring Harb. Perspect. Biol..

[31] Li J., Shang G., Chen Y.-J., Brautigam C.A., Liou J., Zhang X., Bai X.-C. (2019). Cryo-EM analyses reveal the common mechanism and diversification in the activation of RET by different ligands. eLife.

[32] Taraviras S., Marcos-Gutierrez C.V., Durbec P., Jani H., Grigoriou M., Sukumaran M., Wang L.C., Hynes M., Raisman G., Pachnis V. (1999). Signalling by the RET receptor tyrosine kinase and its role in the development of the mammalian enteric nervous system. Development.

[33] Michels S., Scheel A.H., Scheffler M., Schultheis A.M., Gautschi O., Aebersold F., Diebold J., Pall G., Rothschild S., Bubendorf L. (2016). Clinicopathological Characteristics of RET Rearranged Lung Cancer in European Patients. J. Thorac. Oncol..

[34] Zhang K., Chen H., Wang Y., Yang L., Zhou C., Yin W., Wang G., Mao X., Xiang J., Li B. (2019). Clinical Characteristics and Molecular Patterns of RET-Rearranged Lung Cancer in Chinese Patients. Oncol. Res..

[35] Drilon A., Lin J.J., Filleron T., Ni A., Milia J., Bergagnini I., Hatzoglou V., Velcheti V., Offin M., Li B. (2018). Frequency of Brain Metastases and Multikinase Inhibitor Outcomes in Patients With RET–Rearranged Lung Cancers. J. Thorac. Oncol..

[36] Shi M., Wang W., Zhang J., Li B., Lv D., Wang D., Wang S., Cheng D., Ma T. (2022). Identification of *RET* fusions in a Chinese multicancer retrospective analysis by next-generation sequencing. Cancer Sci..

[37] Qiu Z., Ye B., Wang K., Zhou P., Zhao S., Li W., Tian P. (2020). Unique Genetic Characteristics and Clinical Prognosis of Female Patients with Lung Cancer Harboring RET Fusion Gene. Sci. Rep..

[38] Drusbosky L.M., Rodriguez E., Dawar R., Ikpeazu C.V. (2021). Therapeutic strategies in RET gene rearranged non-small cell lung cancer. J. Hematol. Oncol..

[39] Offin M., Guo R., Wu S.L., Sabari J., Land J.D., Ni A., Montecalvo J., Halpenny D.F., Buie L.W., Pak T. (2019). Immunophenotype and Response to Immunotherapy of *RET*-Rearranged Lung Cancers. JCO Precis. Oncol..

[40] Bhandari N.R., Hess L.M., Han Y., Zhu Y.E., Sireci A.N. (2021). Efficacy of immune checkpoint inhibitor therapy in patients with *RET* fusion-positive non-small-cell lung cancer. Immunotherapy.

[41] Seegobin K., Majeed U., Wiest N., Manochakian R., Lou Y., Zhao Y. (2021). Immunotherapy in Non-Small Cell Lung Cancer With Actionable Mutations Other Than EGFR. Front. Oncol..

[42] Meng Y., Yang Y., Fang Y., Lin X., Xie X., Deng H., Wu J., Zhou M., Sun N., Xie Z. (2022). The Treatment Status of Patients in NSCLC With RET Fusion Under the Prelude of Selective RET-TKI Application in China: A Multicenter Retrospective Research. Front. Oncol..

[43] Liu X., Hu X., Shen T., Li Q., Mooers B.H.M., Wu J. (2020). RET kinase alterations in targeted cancer therapy. Cancer Drug Resist..

[44] Ackermann C.J., Stock G., Tay R., Dawod M., Gomes F., Califano R. (2019). Targeted Therapy For RET-Rearranged Non-Small Cell Lung Cancer: Clinical Development And Future Directions. OncoTargets Ther..

[45] Stinchcombe T.E. (2020). Current management of *RET* rearranged non-small cell lung cancer. Ther. Adv. Med. Oncol..

[46] Thein K.Z., Velcheti V., Mooers B.H., Wu J., Subbiah V. (2021). Precision therapy for RET-altered cancers with RET inhibitors. Trends Cancer.

[47] Gautschi O., Milia J., Filleron T., Wolf J., Carbone D.P., Owen D., Camidge R., Narayanan V., Doebele R.C., Besse B. (2017). Targeting RET in Patients With RET-Rearranged Lung Cancers: Results From the Global, Multicenter RET Registry. J. Clin. Oncol..

[48] Platt A., Morten J., Ji Q., Elvin P., Womack C., Su X., Donald E., Gray N., Read J., Bigley G. (2015). A retrospective analysis of RET translocation, gene copy number gain and expression in NSCLC patients treated with vandetanib in four randomized Phase III studies. BMC Cancer.

[49] Lin J.J., Kennedy E., Sequist L.V., Brastianos P.K., Goodwin K.E., Stevens S., Wanat A.C., Stober L.L., Digumarthy S.R., Engelman J.A. (2016). Clinical Activity of Alectinib in Advanced RET -Rearranged Non–Small Cell Lung Cancer. J. Thorac. Oncol..

[50] Ribeiro M.F.S.A., Alessi J.V.M., Oliveira L.J.C., Gongora A.B.L., Sacardo K.P., Zucchetti B.M., Shimada A.K., Barbosa F.D.G., Feher O., Katz A. (2020). Alectinib activity in chemotherapy-refractory metastatic RET-rearranged non-small cell lung carcinomas: A case series. Lung Cancer.

[51] Drilon A., Rekhtman N., Arcila M., Wang L., Ni A., Albano M., Van Voorthuysen M., Somwar R., Smith R.S., Montecalvo J. (2016). Cabozantinib in patients with advanced RET-rearranged non-small-cell lung cancer: An open-label, single-centre, phase 2, single-arm trial. Lancet Oncol..

[52] Nokihara H., Nishio M., Yamamoto N., Fujiwara Y., Horinouchi H., Kanda S., Horiike A., Ohyanagi F., Yanagitani N., Nguyen L. (2019). Phase 1 Study of Cabozantinib in Japanese Patients With Expansion Cohorts in Non–Small-Cell Lung Cancer. Clin. Lung Cancer.

[53] Horiike A., Takeuchi K., Uenami T., Kawano Y., Tanimoto A., Kaburaki K., Tambo Y., Kudo K., Yanagitani N., Ohyanagi F. (2016). Sorafenib treatment for patients with RET fusion-positive non-small cell lung cancer. Lung Cancer.

[54] Lee S.-H., Lee J.-K., Ahn M.-J., Kim D.-W., Sun J.-M., Keam B., Kim T., Heo D., Ahn J., Choi Y.-L. (2017). Vandetanib in pretreated patients with advanced non-small cell lung cancer-harboring RET rearrangement: A phase II clinical trial. Ann. Oncol..

[55] Yoh K., Seto T., Satouchi M., Nishio M., Yamamoto N., Murakami H., Nogami N., Matsumoto S., Kohno T., Tsuta K. (2017). Vandetanib in patients with previously treated RET-rearranged advanced non-small-cell lung cancer (LURET): An open-label, multicentre phase 2 trial. Lancet Respir. Med..

[56] Hida T., Velcheti V., Reckamp K.L., Nokihara H., Sachdev P., Kubota T., Nakada T., Dutcus C.E., Ren M., Tamura T. (2019). A phase 2 study of lenvatinib in patients with RET fusion-positive lung adenocarcinoma. Lung Cancer.

[57] Cascetta P., Sforza V., Manzo A., Carillio G., Palumbo G., Esposito G., Montanino A., Costanzo R., Sandomenico C., De Cecio R. (2021). RET Inhibitors in Non-Small-Cell Lung Cancer. Cancers.

[58] Takamori S., Matsubara T., Haratake N., Toyokawa G., Fujishita T., Toyozawa R., Ito K., Yamaguchi M., Taguchi K., Okamoto T. (2021). Targeted Therapy for RET Fusion Lung Cancer: Breakthrough and Unresolved Issue. Front. Oncol..

[59] Michelotti A., de Scordilli M., Bertoli E., De Carlo E., Del Conte A., Bearz A. (2022). NSCLC as the Paradigm of Precision Medicine at Its Finest: The Rise of New Druggable Molecular Targets for Advanced Disease. Int. J. Mol. Sci..

[60] Singh N., Temin S., Baker S., Blanchard E., Brahmer J.R., Celano P., Duma N., Ellis P.M., Elkins I.B., Haddad R.Y. (2022). Therapy for Stage IV Non-Small-Cell Lung Cancer With Driver Alterations: ASCO Living Guideline. J. Clin. Oncol..

[61] Drilon A., Oxnard G.R., Tan D.S.W., Loong H.H.F., Johnson M., Gainor J., McCoach C.E., Gautschi O., Besse B., Cho B.C. (2020). Efficacy of Selpercatinib in RET Fusion-Positive Non-Small-Cell Lung Cancer. N. Engl. J. Med..

[62] Drilon A., Subbiah V., Gautschi O., Tomasini P., de Braud F., Solomon B.J., Tan D.S.-W., Alonso G., Wolf J., Park K. (2022). Selpercatinib in Patients With *RET* Fusion–Positive Non–Small-Cell Lung Cancer: Updated Safety and Efficacy From the Registrational LIBRETTO-001 Phase I/II Trial. J. Clin. Oncol..

[63] Gainor J.F., Curigliano G., Kim D.-W., Lee D.H., Besse B., Baik C.S., Doebele R.C., Cassier P.A., Lopes G., Tan D.S.W. (2021). Pralsetinib for RET fusion-positive non-small-cell lung cancer (ARROW): A multi-cohort, open-label, phase 1/2 study. Lancet Oncol..

[64] Griesinger F., Curigliano G., Thomas M., Subbiah V., Baik C., Tan D., Lee D., Misch D., Garralda E., Kim D.-W. (2022). Safety and efficacy of pralsetinib in RET fusion–positive non-small-cell lung cancer including as first-line therapy: Update from the ARROW trial. Ann. Oncol..

[65] Fancelli S., Caliman E., Mazzoni F., Brugia M., Castiglione F., Voltolini L., Pillozzi S., Antonuzzo L. (2021). Chasing the Target: New Phenomena of Resistance to Novel Selective RET Inhibitors in Lung Cancer. Updated Evidence and Future Perspectives. Cancers.

[66] Choudhury N.J., Drilon A. (2020). Decade in review: A new era for RET-rearranged lung cancers. Transl. Lung Cancer Res..

[67] Osta B.E., Ramalingam S.S. (2020). RET Fusion: Joining the Ranks of Targetable Molecular Drivers in NSCLC. JTO Clin. Res. Rep..

[68] Solomon B.J., Tan L., Lin J.J., Wong S.Q., Hollizeck S., Ebata K., Tuch B.B., Yoda S., Gainor J.F., Sequist L.V. (2020). RET Solvent Front Mutations Mediate Acquired Resistance to Selective RET Inhibition in RET-Driven Malignancies. J. Thorac. Oncol..

[69] Lin J., Liu S., McCoach C., Zhu V., Tan A., Yoda S., Peterson J., Do A., Prutisto-Chang K., Dagogo-Jack I. (2020). Mechanisms of resistance to selective RET tyrosine kinase inhibitors in RET fusion-positive non-small-cell lung cancer. Ann. Oncol..

[70] Lin J.J., Gainor J.F. (2021). An early look at selective RET inhibitor resistance: New challenges and opportunities. Br. J. Cancer.

[71] Rosen E.Y., Won H.H., Zheng Y., Cocco E., Selcuklu D., Gong Y., Friedman N.D., de Bruijn I., Sumer O., Bielski C.M. (2022). The evolution of RET inhibitor resistance in RET-driven lung and thyroid cancers. Nat. Commun..

[72] Drilon A.E., Zhai D., Rogers E., Deng W., Zhang X., Ung J., Lee D., Rodon L., Graber A., Zimmerman Z.F. (2020). The next-generation RET inhibitor TPX-0046 is active in drug-resistant and naïve RET-driven cancer models. J. Clin. Oncol..

[73] Rosen E.Y., Johnson M.L., Clifford S.E., Somwar R., Kherani J.F., Son J., Bertram A.A., Davare M.A., Gladstone E.G., Ivanova E.V. (2021). Overcoming MET-Dependent Resistance to Selective RET Inhibition in Patients with RET Fusion–Positive Lung Cancer by Combining Selpercatinib with Crizotinib. Clin. Cancer Res..

[74] Nelson-Taylor S.K., Le A.T., Yoo M., Schubert L., Mishall K.M., Doak A., Varella-Garcia M., Tan A.-C., Doebele R.C. (2017). Resistance to RET-Inhibition in RET-Rearranged NSCLC Is Mediated By Reactivation of RAS/MAPK Signaling. Mol. Cancer Ther..

[75] Chang H., Sung J.H., Moon S.U., Kim H.S., Kim J.W., Lee J.S. (2017). EGF Induced RET Inhibitor Resistance in CCDC6-RET Lung Cancer Cells. Yonsei Med. J..

[76] Raman R., Villefranc J.A., Ullmann T.M., Thiesmeyer J., Anelli V., Yao J., Hurley J.R., Pauli C., Bareja R., Eng K.W. (2022). Inhibition of FGF receptor blocks adaptive resistance to RET inhibition in *CCDC6-RET*–rearranged thyroid cancer. J. Exp. Med..

[77] Schubert L., Le A.T., Estrada-Bernal A., Doak A.E., Yoo M., Ferrara S.E., Goodspeed A., Kinose F., Rix U., Tan A.-C. (2021). Novel Human-Derived RET Fusion NSCLC Cell Lines Have Heterogeneous Responses to RET Inhibitors and Differential Regulation of Downstream Signaling. Mol. Pharmacol..

[78] Hayashi T., Odintsov I., Smith R.S., Ishizawa K., Liu A.J.W., Delasos L., Kurzatkowski C., Tai H., Gladstone E., Vojnic M. (2020). RET inhibition in novel patient-derived models of RET fusion- positive lung adenocarcinoma reveals a role for MYC upregulation. Dis. Model. Mech..

[79] Fujimura T., Furugaki K., Harada N., Yoshimura Y. (2020). Enhanced antitumor effect of alectinib in combination with cyclin-dependent kinase 4/6 inhibitor against RET-fusion-positive non-small cell lung cancer cells. Cancer Biol. Ther..

[80] Repetto M., Crimini E., Ascione L., Bielo L.B., Belli C., Curigliano G. (2022). The return of RET GateKeeper mutations? an in-silico exploratory analysis of potential resistance mechanisms to novel RET macrocyclic inhibitor TPX-0046. Investig. New Drugs.

[81] Drilon A., Rogers E., Zhai D., Deng W., Zhang X., Lee D., Ung J., Whitten J., Zhang H., Liu J. (2019). TPX-0046 is a novel and potent RET/SRC inhibitor for RET-driven cancers. Ann. Oncol..

[82] Di Noia V., D’Aveni A., D’Argento E., Rossi S., Ghirardelli P., Bortolotti L., Vavassori V., Bria E., Ceresoli G. (2021). Treating disease progression with osimertinib in EGFR-mutated non-small-cell lung cancer: Novel targeted agents and combination strategies. ESMO Open.

[83] Pan Y., Deng C., Qiu Z., Cao C., Wu F. (2021). The Resistance Mechanisms and Treatment Strategies for ALK-Rearranged Non-Small Cell Lung Cancer. Front. Oncol..

[84] Yu H., Goldberg S., Le X., Piotrowska Z., Smith P., Mensi I., Kirova B., Chmielecki J., Li-Sucholeicki X., Szekeres P. (2019). P2.01-22 ORCHARD: A Phase II Platform Study in Patients with Advanced NSCLC Who Have Progressed on First-Line Osimertinib Therapy. J. Thorac. Oncol..

